# Microbial sulfate reduction and metal attenuation in pH 4 acid mine water

**DOI:** 10.1186/1467-4866-8-10

**Published:** 2007-10-23

**Authors:** Clinton D Church, Richard T Wilkin, Charles N Alpers, Robert O Rye, R Blaine McCleskey

**Affiliations:** 1 U.S. Geological Survey, California Water Science Center, 4165 Spruance Road, San Diego, CA 92101, USA; 2 U.S. Department of Agriculture, Agricultural Research Service, Curtin Road, Building 3702, University Park, PA 16802, USA; 3 U.S. Environmental Protection Agency, National Risk Management Research Laboratory, 919 Kerr Research Drive, Ada, OK 74820, USA; 4 U.S. Geological Survey, California Water Science Center, 6000 J Street, Sacramento, CA 95819, USA; 5 U.S. Geological Survey, Denver Federal Center, Mailstop 963, Denver, CO 80225, USA; 6 U.S. Geological Survey, Suite 127, 3215 Marine Street, Boulder, CO 80303, USA

## Abstract

Sediments recovered from the flooded mine workings of the Penn Mine, a Cu-Zn mine abandoned since the early 1960s, were cultured for anaerobic bacteria over a range of pH (4.0 to 7.5). The molecular biology of sediments and cultures was studied to determine whether sulfate-reducing bacteria (SRB) were active in moderately acidic conditions present in the underground mine workings. Here we document multiple, independent analyses and show evidence that sulfate reduction and associated metal attenuation are occurring in the pH-4 mine environment. Water-chemistry analyses of the mine water reveal: (1) preferential complexation and precipitation by H_2_S of Cu and Cd, relative to Zn; (2) stable isotope ratios of ^34^S/^32^S and ^18^O/^16^O in dissolved SO_4 _that are 2–3 ‰ heavier in the mine water, relative to those in surface waters; (3) reduction/oxidation conditions and dissolved gas concentrations consistent with conditions to support anaerobic processes such as sulfate reduction. Scanning electron microscope (SEM) analyses of sediment show 1.5-micrometer, spherical ZnS precipitates. Phospholipid fatty acid (PLFA) and denaturing gradient gel electrophoresis (DGGE) analyses of Penn Mine sediment show a high biomass level with a moderately diverse community structure composed primarily of iron- and sulfate-reducing bacteria. Cultures of sediment from the mine produced dissolved sulfide at pH values near 7 and near 4, forming precipitates of either iron sulfide or elemental sulfur. DGGE coupled with sequence and phylogenetic analysis of 16S rDNA gene segments showed populations of *Desulfosporosinus *and *Desulfitobacterium *in Penn Mine sediment and laboratory cultures.

## Introduction

Acid mine drainage (AMD) is caused primarily by the oxidation of sulfide minerals and is characterized by high aqueous concentrations of metals and low pH values in the absence of neutralizing agents such as carbonates. Although the oxidation of sulfide minerals can be abiotic, the oxidation rate can be enhanced by several orders of magnitude by sulfur- and iron-oxidizing bacteria [1-3] and archaea [4].

Sulfate-reducing bacteria (SRB), together with metal-reducing bacteria, have the ability to reverse the reactions causing acid mine drainage, attenuating metal concentrations by precipitation of sulfide minerals [*e.g*. [5]], and raising the pH of the water [6]. The overall sulfate-reduction process can be summarized as:

2CH_2_O_(aq) _+ SO_4_^2-^_(aq) _+ 2H^+^_(aq) _⇒ H_2_S_(aq) _+ CO_2(aq) _+ H_2_O_(l) _

where CH_2_O_(aq) _represents dissolved organic carbon. The resulting sulfide can precipitate with divalent metals in AMD, for example (M = Cd, Cu, Fe, Ni, Pb, or Zn):

H_2_S_(aq) _+ M^2+^_(aq) _⇔ MS_(s) _+ 2H^+^_(aq) _

The mass concentration of reactants involved in equation (1) is usually much larger than the mass concentration of metals in equation (2), therefore this process can lead to an increase in the alkalinity and pH value of the water, while simultaneously attenuating divalent metals. A number of bacterial consortia have been shown to mediate this reaction, which is most commonly observed to occur at circum-neutral pH; most sulfate-reducing bacteria have been considered to be inactive at pH < 5 [7-9]. However, recent studies of acid mine drainage systems (both engineered and natural) have noted that there is some potential for low-pH sulfidogenesis [10-15]. Here we document multiple, independent analyses and show evidence that bacterial sulfate reduction, metal attenuation, and thus, partial natural acid mine remediation, can occur in the moderately acidic, pH 4 environment.

In laboratory studies, sulfate reduction has been shown to occur in solutions as low as pH 3 in column and fixed-bed bioreactors using ethanol, methanol, or glycerol (alone or in various combinations) as an organic substrate [10,15]. A highly controlled fermenter study showed preferential ZnS precipitation while ferrous iron remained in solution if cultures were maintained at pH 3.8–4.2 [12]. Analysis of the 16S rRNA gene in two of these studies showed that the acidophillic SRB were related to *Desulfosporosinus orientus *[12,13].

In-situ remediation of metals by sulfate reduction has been shown to occur in acidic pit lakes and sediments after the pH was raised to 5–6 by amendment with carbokalk (a waste product from the sugar industry, containing organic carbon and lime) [16-19]. Koschorreck et al. [20] discussed SRB activity at pH ~3 in sediments beneath a volcanically-acidified lake. In natural AMD systems, the reduction of sulfate to sulfide has been reported at pH values as low as 2–3 [17,21-23]. In a recent study, Roesler et al. [24] report low levels of aqueous sulfide at circum-neutral pH in certain flooded underground mine workings of Butte Montana. However, there are few reports of the isolation and/or characterization of acidophilic SRB from these mine-impacted environments. In one report, two sulfate-reducing bacteria isolates related to *Desulfosporosinus orientus *were capable of reducing sulfate in the pH range of 4.9–6.1, with an optimum pH of 5.5 [13,23], and Sen [21] described SRB that also appear to be related to *Desulfosporosinus *species and that grow at pH values as low as 3.

### Site history and background

The Penn Mine, located in Calaveras County, California, operated from 1861 to 1953 and produced nearly 900,000 metric tons of ore, the most of any deposit in the Foothill Cu-Zn belt of massive sulfide deposits [25,26]. Acidic, metal-rich surface runoff from Penn Mine, primarily from oxidation of sulfide minerals in surface waste piles, affected the Mokelumne River basin after mining began in the 1860's. Fish kills were documented in the Mokelumne River in the 1930's. Camanche Reservoir was completed in 1963, flooding the reach of the Mokelumne River adjacent to the Penn Mine. Impoundments on the mine site, built to improve water quality in the late 1970's, were insufficient to prevent periodic discharges of acidic, metal-rich water to the reservoir. In the late 1990's, as part of a legal settlement, $10 million was invested to restore the Penn Mine site by moving approximately 290,000 cubic meters of waste material to a lined, on-site landfill; all impoundments were removed, natural stream channels and surface water flow were restored, and more than 10 hectares of riparian habitat were enhanced. Following this restoration, the annual loads of copper and zinc from the Penn Mine were reduced by 9,000 kg (99%) and 36,000 kg (92%), respectively [27].

The remediation of surface waste piles did not affect the deep mine workings at Penn Mine, which still contain acidic, metalliferous water. Seepage of metal-rich water continued in Hinkley Run, an area where shafts and adits connect the deep mine workings to the surface [28,29]. Spatial and temporal variations in water quality relative to metal inputs from the seepage area, and the soil chemistry and phytoaccumulation of Cu and Zn in native grasses and a legume have been described [27,30].

Previous work on the geochemistry of ground water in the underground workings of the Penn Mine indicated that sulfate reduction is likely to be occurring [28,29]. Evidence for active sulfate reduction was three-fold: (1) ratios of Zn/Cu and Zn/Cd in the mine water were anomalously high compared with nearby surface waters, consistent with preferential scavenging by H_2_S of Cu and Cd relative to Zn; (2) stable isotope ratios of ^34^S/^32^S and ^18^O/^16^O in dissolved SO_4 _were 2–3 ‰ heavier in the mine water relative to surface waters, consistent with preferential reduction of ^32^S and ^16^O by SRB [[31] and references within]; and (3) dissolved gases in the mine water were O_2_-poor with traces of H_2 _and CH_4_, consistent with redox conditions that would favor anaerobic microbes such as SRB. The pH of the mine water in 1991–92 was 4.0, substantially lower than the pH range of 5.5–8.0 typically cited for sulfate-reducing bacteria [7].

The purpose of the current investigation was to determine whether SRB are active in the Penn Mine ground water at pH values near 4. An alternative hypothesis is that sulfate reduction takes place in microenvironments at pH values in the range of 5.5–8.0, contributing H_2_S to a mine pool with an overall pH of about 4. To determine whether microbially mediated sulfate reduction could occur at pH 4, we cultured microbial samples from sediments collected from the mine workings over a range of pH values, and investigated the molecular biology of the cultures to evaluate whether or not known SRB were present.

### Experimental design

#### Sediment collection

Sediments were collected aseptically from the mine floor using a large-water-volume, depth-dependent bailer system designed for the task [see Additional file 1 for an image recorded using a down-hole camera during sample retrieval from the mine floor]. Sediments were collected in November 2001 and November 2002. The bailer system was deployed 4 times and approximately 2 g of sediment in total were collected from the mine workings. Limited sample mass precluded duplicate analyses of most of the solid-phase tests. Based on observations using a down-hole camera, the bailer device collected sediments that appeared to be representative of the subsurface environment. Sampling chambers were opened in a Coy anaerobic glovebox. Sample splits were dried anaerobically for subsequent sediment characterization analyses. For microbial analyses, sample splits were frozen until DNA isolation.

#### Sediment characterization

Total sulfur and organic carbon concentrations in sediment were determined coulometrically [32,33]. Temperatures of combustion were 1100°C for sulfur and 950°C for carbon. Powder X-ray diffraction scans were collected using a Rigaku MiniFlex at 30 kV, 15 mA and with Cu*K*α_1 _radiation. Samples were smeared on a zero-background quartz plate and scanned from 5 to 90° (2θ). Scanning electron microscopy analyses were conducted using a LEO model 982 microscope with backscatter preamplifier and an Oxford model 6901 energy-dispersive x-ray spectrophotometer at the USGS laboratory in Menlo Park, CA (M.P. Hunerlach and R.C. Oscarson, analysts).

#### Water sampling and analysis

Water samples were collected from discrete depths from borehole GS-18, which intersects a stope on the 300 level of the Penn Mine [27,28]. Depths sampled in November 2001 were 33.0 m, 75.0 m, 78.0 m, and 79.3 m below land surface. In November 2002, the following depths were sampled: 75.0 m, 78.0 m, 79.3 m, and 81.0 m (immediately above the sediment-water interface). A Fultz pump equipped with a Teflon-lined sampling tube was used for collecting water samples.

Prior to sample collection, a multi-parameter probe was inserted into the borehole and measurements were made of pH, specific conductance, and temperature at various depths. During pumping from each discrete depth from which a water sample was collected, a flow-through chamber with probe inserts was used to monitor pH, specific conductance, temperature, oxidation-reduction potential [ORP, converted to Eh; see Additional file 2], and dissolved oxygen of the water. Measured ORP values were converted to Eh values by adding the difference between the measured ORP of the reference solution and the theoretical ORP of the reference solution. In 2002, dissolved oxygen was measured with a CHEMetrics colorimetric kit (Rhodazine D).

Water samples were filtered using tortuous-path Gelman capsule filters with 0.45-μm nominal pore diameter. Concentrations of anions (F, Br, Cl, and SO_4_) were determined by ion chromatography (IC) [34]. Sulfate concentrations in the 2001 samples were determined by the USEPA laboratory in Ada, OK; the 2002 anion samples were analyzed by the USGS laboratory in San Diego, CA. Fluoride, Br, and Cl concentrations were analyzed by the USGS laboratory in Boulder, CO. Concentrations of major cations (Ca, K, Na, Mg, Si) and selected trace elements (Al, B, Ba, Be, Cd, Co, Cu, Mn, Ni, Pb, Se, Sr, V, and Zn) in water were determined by inductively coupled plasma – atomic emission spectrometry (ICP-AES) at the USGS laboratory in Boulder, CO. Redox species of arsenic and iron were determined at the USGS laboratory in Boulder, CO. Arsenic (III) and arsenic (total) were determined by hydride generation atomic absorption spectrometry (HGAAS) [35]. Iron (II) and iron (total) were determined using colorimetric methods with FerroZine [36]. McCleskey et al. [37] describe the IC, ICP-AES, HGAAS, and Fe colorimetric methodology used at the USGS laboratory in Boulder, CO. For all analyses, environmental samples were diluted as necessary to bring the analyte concentration within the optimal range of the method. Each sample was analyzed at least twice for each dilution and for all constituents. There is frequently an optimum dilution which produces the most accurate value, but comparison of values produced by the various dilutions serves as an additional accuracy check. The dilutions were prepared with double-distilled or deionized water and re-distilled or trace metal grade acids [see Additional file 1 and 2 for information on quality assurance and quality control of water analyses].

Stable isotope ratios of oxygen (^18^O/^16^O) and hydrogen (^2^H/^1^H, where ^2^H is deuterium or D) in water were determined in the laboratory at the Department of Geology, University of California, Davis, under the direction of Howard Spero. Oxygen isotope ratios in water, expressed as δ^18^O_H2O _in units of per mil (‰, parts per thousand) relative to Vienna Standard Mean Ocean Water (VSMOW), were determined by conventional isotope ratio mass spectrometry after equilibration with carbon dioxide, using a modification of the technique of Epstein and Mayeda [38]. Hydrogen isotope ratios, expressed as δD in units of per mil relative to VSMOW, were determined on a MAT-252 spectrometer using the platinum rod technique [39]. Three working standards calibrated to VSMOW and standard reference waters supplied by the International Atomic Energy Agency (IAEA) were analyzed in duplicate with each batch of water samples analyzed. Analytical uncertainty was ± 0.1 ‰ for δ^18^O_H2O _and ± 1 ‰ for δD. Absolute deviations of replicate samples were within 0.1 ‰ for δ^18^O_H2O _and within 1 ‰ for δD [see Additional file 2].

Stable isotope ratios of sulfur (^34^S/^32^S) and oxygen (^18^O/^16^O) in aqueous sulfate were determined at the USGS laboratory in Denver, CO, using continuous-flow isotope-ratio mass spectrometry techniques [40,41]. Sulfur isotopes in aqueous sulfate are expressed as δ^34^S_SO4 _and are reported relative to the Cañon Diablo Troilite (CDT). Oxygen isotopes in aqueous sulfate are expressed as δ^18^O_SO4 _and are reported relative to VSMOW. Analytical uncertainty was ± 0.2 ‰ for δ^34^S_SO4 _and ± 0.5 ‰ for δ^18^O_SO4_. Absolute deviations of replicate samples were within 0.1 ‰ for δ^34^S_SO4 _and δ^18^O_SO4 _[see Additional file 2]. Stable isotope ratios of carbon (^13^C/^12^C) were determined at the USGS laboratory in Menlo Park, CA and are reported relative to the Vienna Pee Dee Belemnite (VPDB).

Samples for analysis of dissolved gas concentrations Ar, CH_4_, C_2_H_6_, CO_2_, H_2_, and N_2 _were collected under ambient pressure using glass side-arm samplers. Dissolved gas concentrations were measured by gas chromatography (GC) at the USGS laboratory in Menlo Park, CA (W.C. Evans, analyst) [42].

#### Microbial characterization

Samples from the Penn Mine sediments were shipped frozen to Microbial Insights (Rockford, TN) for analysis of phospholipid fatty acids (PLFA) and denaturing gradient gel electrophoresis (DGGE). Frozen sediment was extracted with a single-phase chloroform-methanol-phosphate buffer at room temperature for 3 h [43]. The organic phase containing bacterial lipids was collected and fractionated onto silicic acid columns into glycolipids, neutral lipids, and polar lipids. Lipid fractions were analyzed and quantified using capillary GC with structural analysis by gas chromatography-mass spectrometry (GC-MS). DGGE analysis followed molecular techniques described elsewhere [44,45]. In this method, sample microbial DNA is isolated and purified using organic extraction and precipitation techniques [46]. Samples were mixed with sodium phosphate buffer and glass beads and agitated in a microcentrifuge tube using a high-speed bead beater. Chloroform was added, mixed thoroughly, and the tube was recentrifuged. The aqueous supernatant was collected and extracted with phenol/chloroform/isoamyl alchohol (24:24:1). Glycogen was added and the DNA was precipitated from the aqueous phase with an equal volume of isopropanol. The DNA sequence for bacterial 16S ribosomal RNA (rDNA) was then amplified using the polymerase chain reaction (PCR). Separation in DGGE is based on the electrophoretic mobility of partially melted DNA molecules in polyacrylamide gels [44]. The primers targeted eubacterial 16S rDNA regions corresponding to *E. coli *positions 341–534. A portion (20%) of each PCR product was analyzed by agarose gel electrophoresis and ethidium bromide fluorescence. The amount of DNA used for DGGE was standardized to approximately 200 ng by comparison to molecular weight standards. DGGE was performed using a D-Code 16/16 cm gel system maintained at a constant temperature of 60°C in 6 L of buffer solution (20 mM Tris acetate, 0.5 mM EDTA, pH 8.0). Phylogenetic affiliations for each sequenced DNA band were assigned by comparison to known bacterial sequences recorded in the National Center for Biotechnology Information database [GenBank, [47]]. Phylogenetic information was obtained by comparing the measured sequences to the sequences deposited in the GenBank DNA database by using the BLAST algorithm [48].

#### Microcosm experiments

Bacterial cultures were prepared using sediments collected from the Penn Mine workings in an anaerobic chamber under a mixed N_2_:H_2 _gas atmosphere (95%:5%). Approximately 150 mg of sediment was loaded into a 60-mL glass vial along with a volume of fresh growth medium. Vials were capped with butyl rubber stoppers and aluminum crimp seals. Headspace volume was negligible. The nutrient medium composition was ammonium sulfate ((NH_4_)_2_SO_4_), 2500 mg L^-1^; sodium sulfate (Na_2_SO_4_), 1800 mg L^-1^; sodium chloride (NaCl), 2200 mg L^-1^; calcium sulfate (CaSO_4_), 60 mg L^-1^; potassium phosphate (KH_2_PO_4_), 50 mg L^-1^; sodium acetate (CH_3_COONa), 6,000 mg L^-1^; sodium citrate (Na_3_C_6_H_5_O_7_), 300 mg L^-1^; magnesium sulfate (MgSO_4_), 60 mg L^-1^; and ferrous sulfate (FeSO_4_), 12 mg L^-1^. The phosphate concentration was selected to be lower in the growth medium compared to traditional Postgate's media to prevent precipitation of insoluble calcium, aluminum, or ferric iron phosphates. The pH of the medium was adjusted to 7.00 ± 0.15. Initial cultures were established in undiluted growth media and in media diluted to 10% with deionized water sterilized by autoclaving at 110°C for 2 h. Activity of sulfate-reducing bacteria was evident after approximately 1 week, based on the appearance of black precipitates of iron sulfide.

Low-pH and near-neutral pH cultures were next established by spiking the primary cultures into pH-adjusted growth media. Acidic growth medium was prepared by additions of 1 N H_2_SO_4_. Sterile control experiments were performed to differentiate biotic and abiotic processes. At selected time intervals, sub-samples were withdrawn by syringe for analyses of pH, ORP, and total dissolved sulfide. Total dissolved sulfide was determined spectrophotometrically by the methylene blue method [49]. After 1 month, the cultures were retrieved and characterized by DGGE.

## Results and discussion

### Sediment characterization

**Figure 1:**
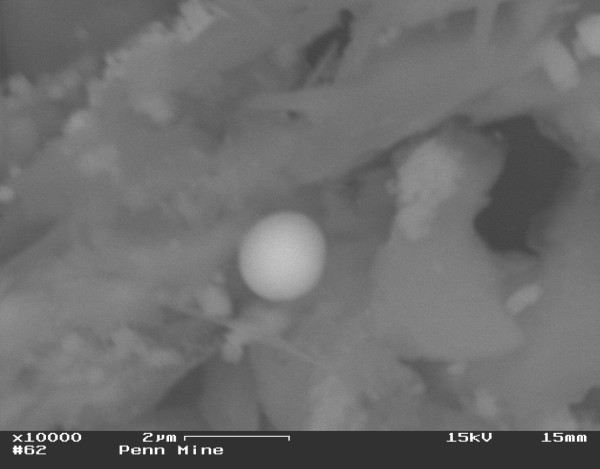
Zinc sulfide spherule precipitated on the surface of what may have been a bacterium.

Total sulfur content in the Penn Mine sediment was determined to be 6.06 ± 0.12 wt%. Approximately 7% of the total sulfur was extracted in a dilute bicarbonate-carbonate buffer (pH 10, extraction carried out under anoxic conditions), a sulfur fraction associated with soluble sulfates. Total organic carbon concentration was 0.85 ± 0.05 wt%. Powder X-ray diffraction indicated the dominant presence of quartz and muscovite with minor amounts of pyrite in the mine sediments. Based on the identification of pyrite in the diffraction patterns it is likely that most of the total sulfur content is associated with sulfides, pyrite in particular. SEM analysis of the sediments (Figure 1) revealed the presence of spheroidal ZnS precipitates. An hypothesis for the formation of similar ZnS spheroids in an underground mine environment with near-neutral pH (a carbonate-hosted Pb-Zn deposit in Wisconsin, USA) was presented by Labrenz et al. [50] and Labrenz and Banfield [51], who considered nanometer-sized ZnS grains in bacterial slime of SRB to be produced by aerotolerant microbes as a mechanism for H_2_S regulation. Concentric banding within larger (μm-scale) spheroidal aggregates of ZnS grains was noted by Moreau et al. [52]. Geochemical models for formation of low-temperature Cu-Pb-Zn deposits have been proposed, based on metal fixation of H_2_S produced by SRB [53].

### Trace metals

The trace-element chemistry of water samples pumped from the deep mine workings during 1991–93 [26,27] and during 2001–02 (this study) shows the effects of sulfate reduction. Acidic surface waters and shallow ground waters at Penn Mine have had consistently elevated concentrations of Zn (100 to 1000 mg L^-1^), Cu (10 to 100 mg L^-1^), and Cd (0.2 to 3.0 mg L^-1^). Deep mine waters have had Zn concentrations in a similar range (10 to 400 mg L^-1^), but much lower concentrations of Cu (< 0.003 to 0.3 mg L^-1^) and Cd (0.01 to 0.05 mg L^-1^). Table 1 presents a summary of water chemistry results from deep mine waters collected from well GS-18. Ratios of Zn/Cu and Zn/Cd in the deep mine waters were consistently higher than those in the surface waters and shallow ground waters. The order of solubility of metal sulfides (most soluble to least soluble) is MnS > FeS > NiS-ZnS > CdS-PbS > CuS > HgS [54]. Therefore, CdS and CuS are less soluble than ZnS, so that Cd and Cu would be preferentially scavenged over Zn by any H_2_S produced by sulfate reduction. Thus, after Cu and Cd are depleted by reaction with H_2_S, additional H_2_S production would result in precipitation of ZnS, which is consistent with the ratios observed in this study. An alternative hypothesis is that Cu and Cd attenuation processes follow other pathways, such as mineral replacement. Previously formed sulfides, such as pyrite, could act as reactants in replacement reactions involving metals that form highly insoluble sulfide precipitates.

**Table 1:** Summary of water chemistry from the 75 to 81 m depth range of well GS-18, Penn Mine, California, 2001–2002 [see Additional file 2].

**Parameter**	**range of values**
Temperature (°C)	20–21
pH	3.90–4.27
Eh (V)	0.35–0.51
Specific Conductance (mS/cm)	3.55–3.74
Al (mg L^-1^)	4.9–8.9
Na	51–63
K	12–17
Mg	90–110
Ca	440–500
Fe_T_	526–586
Fe(II)	521–584
Mn	6.9–8.2
Cd	0.013–0.039
Cu	<0.003–0.01
Zn	10–13
As_T_	0.048–0.121
As(III)	0.035–0.112
Cl	9.7–17
SO_4_	2,580–2,900
DOC	1.8–2.2

### Dissolved gases and redox conditions

Concentrations of dissolved gasses measured in the mine waters during 1991–94 [28] and 2001–02 were consistent with mildly reducing conditions (anoxic to suboxic) generally associated with the active biogenic sulfate reduction [*e.g*. [50,51]]. Dissolved oxygen (DO) concentrations at depths 75.0 to 79.3 m below surface in 2002, determined using a CHEMetrics colorimetric kit, were 0.05 to 0.3 mg L^-1^. The 2002 DO results are considered more reliable than the 2001 measurements that used a flow-through cell, which gave results of 0.5 to 1.1 mg L^-1 ^[see Additional file 2]. Other dissolved gas concentrations (in units of mg L^-1^) were: Ar 0.64 to 1.08; CH_4 _0.18 to 0.42; C_2_H_6 _0.001 to 0.002; CO_2 _704 to 1,320; H_2 _<0.02 to 3.4; and N_2 _14.8 to 28.8. Dissolved H_2 _concentrations of 0.001 to 0.003 μg L^-1 ^have been reported in association with SRB at circum-neutral pH [[55], and references within]. At lower pH it is possible that higher dissolved H_2 _concentrations are able to accumulate due to slower rates of sulfate reduction.

Direct measurements of ORP (by platinum electrode) indicated Eh values from 0.35 to 0.51 V in the deep (≥ 75 m) mine ground water (Table 1), whereas Eh values from speciation calculations based on measured concentrations of Fe(II) and Fe(total) ranged from 0.51 to 0.55 V. The general range of Eh commonly associated with SRB is -0.3 to 0.0 V at circum-neutral pH [55]. At lower pH values, sulfate-reducing environments would be expected to be associated with more positive oxidation-reduction potentials. Although some SRB are aerotolerant, it is possible that they are active in microenvironments that are more reducing than the general mine pool chemistry (analogous to microenvironments in oceanic sediments). It is also possible that sediment pore-water pH and Eh conditions are distinctive from the overlying water column.

### Stable isotopes

The δ^13^C of dissolved inorganic carbon in a Penn mine water sample collected in 2001 was -17 ‰ (VPDB). Oxidative decay of organic matter (such as mine timber) is a likely source of the organic carbon, which is converted to CO_2 _by microbial oxidation. Atmospheric CO_2 _has a δ^13^C value of -7 ‰ (VPDB). C3 plants (e.g., trees and most other plant species) typically have δ^13^C values from -32 to -20 ‰ (average -28.1 +/- 2.5 [56]. The δ^13^C value of -17 ‰ may be a combination of carbon from C3 plants typically used for mine timber (*e.g*. eucalyptus and pine) mixed with atmospheric CO_2_. There may also be a contribution from dissolution of carbonate minerals that may have persisted in host rocks with relatively weak hydrothermal alteration. Although no carbon isotope data are available for carbonate minerals from Penn Mine, such minerals from similar Kuroko-type massive sulfide deposits generally have δ^13^C values ranging from about -7 ‰ VPDB, corresponding to igneous carbonates, to about 0 ‰ VPDB, corresponding to marine carbonates [57,58].

**Figure 2:**
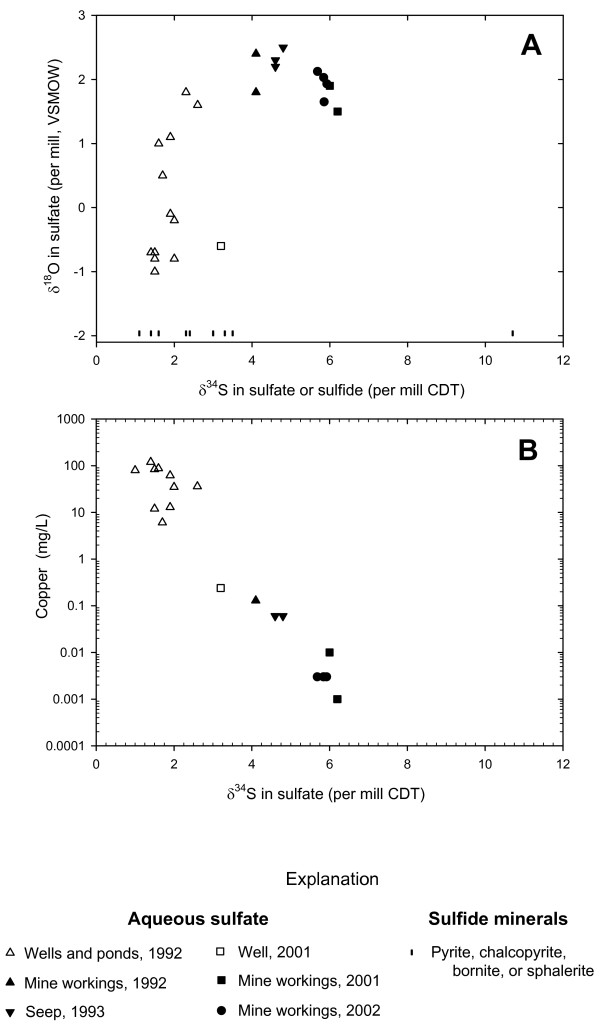
Plots of stable isotopes of aqueous sulfate. A) δ^34^S_SO4 _*vs*. δ^18^O_SO4_; δ^34^S of sulfide minerals shown as vertical bars; B) δ^34^S_SO4 _*vs*. dissolved copper concentration. Data points with error bars indicate non-detects for copper, for which symbols plotted at one-half of the method detection limit (MDL) and error bars extend upward to the MDL (0.003 mg L^-1^). Enrichment of δ^34^S_SO4 _in residual mine pool is likely caused by preferential reduction of ^32^SO_4 _by sulfate-reducing bacteria, producing H_2_S that scavenges aqueous copper from solution.

The δ^34^S and δ^18^O of dissolved sulfate are useful tracers with regard to origins of sulfate and in some situations can be used to track sulfate-reduction processes [59-61]. Deep mine waters at Penn Mine had δ^34^S_SO4 _values of 4 to 7 ‰ (VCDT) whereas those of shallow mine waters (surface waters and shallow wells) ranged from 1 to 3 ‰, similar to the range of δ^34^S in 10 of 11 analyses of primary sulfide minerals (Figure 2A). A similar difference of about 3 ‰ was seen in values of δ^18^O_SO4_, with larger values in the deep mine workings. We interpret this pattern as a shift in δ^34^S and δ^18^O of dissolved sulfate in the residual mine pool caused by microbial sulfate reduction. Microbes preferentially reduce ^32^S and ^16^O relative to ^34^S and ^18^O in the sulfate molecule [59]. A shift of 3 ‰ in δ^34^S corresponds to reduction of about 5% of the sulfate pool in a closed system, based on Rayleigh fractionation [31]. Concentrations of dissolved copper in samples of mine water were found to show a systematic decrease with increasing δ^34^S (Figure 2B), which we attribute to scavenging of Cu from solution by H_2_S produced by sulfate reduction, resulting in precipitation of secondary Cu sulfides. Once Cu, Cd, and Pb have been quantitatively depleted in the mine pool, it is expected that continued H_2_S production would scavenge Zn as ZnS. An alternative explanation for the δ^34^S and δ^18^O trends of dissolved sulfate involves dissolution of primary sulfate minerals such as anhydrite, which occurs in other massive sulfide deposits. This explanation, however, is unlikely given the lack of anhydrite and gypsum in the mine waste piles [62] and the fact that gypsum is only a minor gangue constituent at Penn Mine [63].

### PLFA extracts

Detection of signature lipid biomarkers is an effective means of detecting general groupings of microorganisms in environmental matrices [43]. Biomass concentration in the Penn Mine sediment based on the extraction of phospholipid fatty acids (PLFA) was about 35,000 pmoles g^-1^, equivalent to about 7.1 × 10^8 ^cells g^-1 ^[62]. A comparison is shown in Table 2 of the relative percentages of total PLFA structural groups. Normal saturates are ubiquitous in microbiota; terminally branched saturates (TerBrSats) are attributed to Gram-positive bacteria and some anaerobic Gram-negative bacteria [43]. Branched monoenoic fatty acids are found in anaerobic, metal-reducing bacteria and mid-chain branched fatty acids are common in metal-reducing and anaerobic sulfate-reducing bacteria. Monoenoic fatty acids are found in Gram-negative bacteria and polyenoic fatty acids are found in eukaryotic organisms. PLFA results for material collected from the Penn Mine suggest the presence of a moderately diverse community structure composed of both Gram-negative and Gram-positive bacteria. The Penn Mine sample also was enriched in PLFA structures associated with sulfate-reducing bacteria (Table 2).

**Table 2:** Results of phospholipid fatty acid (PLFA) analysis of Penn Mine sediment.

Total biomass	35,100 pmoles g^-1^
Cell equivalent value^1^	7.08 × 10^8^
Bacterial biomass	31,900 pmoles g^-1^
Eukaryotic biomass	3,230 pmoles g^-1^
ratio bacteria/eukarya	10
Community Structure	% PLFA
Gram+/anaerobic Gram- (TerBrSats)	9.0
Gram- (Monoenoic)	36.8
Anaerobic metal reducers (Branched monoenoic)	0.0
SRB (Mid-chain branched)	18.2
Genera (Normal saturates)	26.8
Eukaryotes (polyenoics)	9.2

^1 ^The cell equivalent value is calculated from experiments with typical bacteria isolated from soil and water [62]. This value is based on 2.0 × 10^12 ^cells per gram dry weight of cells and 10^8 ^picomoles of phospholipid per gram dry weight of cells. The number of cells per gram of dry weight may vary and is dependent on the environmental conditions from which the microorganisms were recovered.

### DGGE results

**Figure 3:**
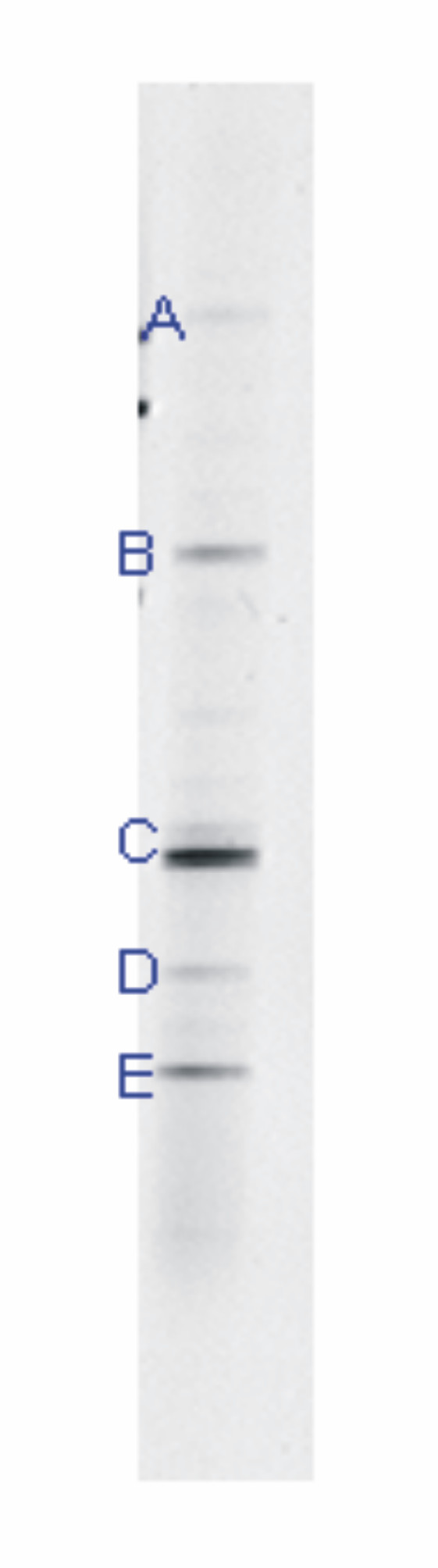
DGGE gel image of 16S rDNA fragments from Penn Mine sediments with five bands labeled (Table 3, sample collected November 2001).

Denaturing gradient gel electrophoresis (DGGE) analysis was used to determine the dominant bacteria in a sample from the Penn Mine (Figure 3 and Table 3). The bacterial profile for Penn Mine sediment contained five bands that were excised and sequenced. Bands A, B, and C were all associated with bacteria within the *Clostridia *group (Figure 3, Table 3). Specifically, bands A and B were associated with the Gram-positive, metal-reducing bacteria *Desulfosporosinus *and *Desulfitobacterium*. Members of the genus *Desulfosporosinus *are spore-forming, sulfate-reducing bacteria that have been found in gasoline-contaminated ground water [64] and in low pH sulfidogenic reactor systems [14]. *Desulfitobacterium *are anaerobic bacteria that have been shown to reductively dechlorinate perchloroethylene, trichloroethylene, and chlorophenol compounds. *Desulfitobacterium *species have also been shown to reduce sulfite, thiosulfate, and sulfur to sulfide, as well as reducing nitrate to nitrite [65]. The sequence obtained from band C aligned with an unidentified eubacterium clone BSV24, which was also affiliated with the *Clostridia *group [66].

**Table 3:** Sequence results from DGGE bands excised from Penn Mine sediment (Figure 3). Sequence identifications were performed using the BLASTN facility of the National Center for Biotechnology Information [47].

**Band**	**Closest Match**	**Similarity Index**	**Phylogenetic Affiliation**
A	*Desulfosporosinus *sp.	98%	Clostridia
B	*Desulfitobacterium *sp.	92%	Clostridia
C	Uncultured bacterium	92%	Clostridia
D	Uncultured eubacteria *Geothrix fermentans*	94% 97%	Nitrospina
E	Unsequencable	-	-

Band D was most closely associated with an uncultured bacterium that has previously been identified from a landfill site where iron reduction occurred [67]. The next closest affiliation was with *Geothrix fermentans *based on the similarity index. Members of *Geothrix fermentans *are strict anaerobes that conserve energy to support growth from the complete oxidation of organic acids to carbon dioxide, with Fe(III) serving as the sole electron acceptor. These bacteria also have the ability to use alternative electron acceptors such as Mn(IV), nitrate, fumarate, and the humic acid analog 2,6-anthraquinone disulfonate, or can grow fermentatively with some intermediates of the citric acid cycle as the substrate. *Geothrix fermentans *and closely related strains have been recovered from the Fe(III) reduction zone of petroleum-contaminated aquifers [67,68]. *Geothrix *species are often enriched in aquifer sediments when Fe(III) reduction becomes an important terminal electron-accepting process. These bacteria-release compounds that can serve as electron shuttles and can solubilize Fe(III) from Fe(III) oxide. Thus, this dissimilatory Fe(III)-reducing microorganism does not need to contact insoluble Fe(III) oxide directly to reduce Fe(III) [68]. B and E failed to produce a useable sequence; the DNA sequence was not closely related to any of those in the GenBank database.

### Bacterial cultures

**Figure 4:**
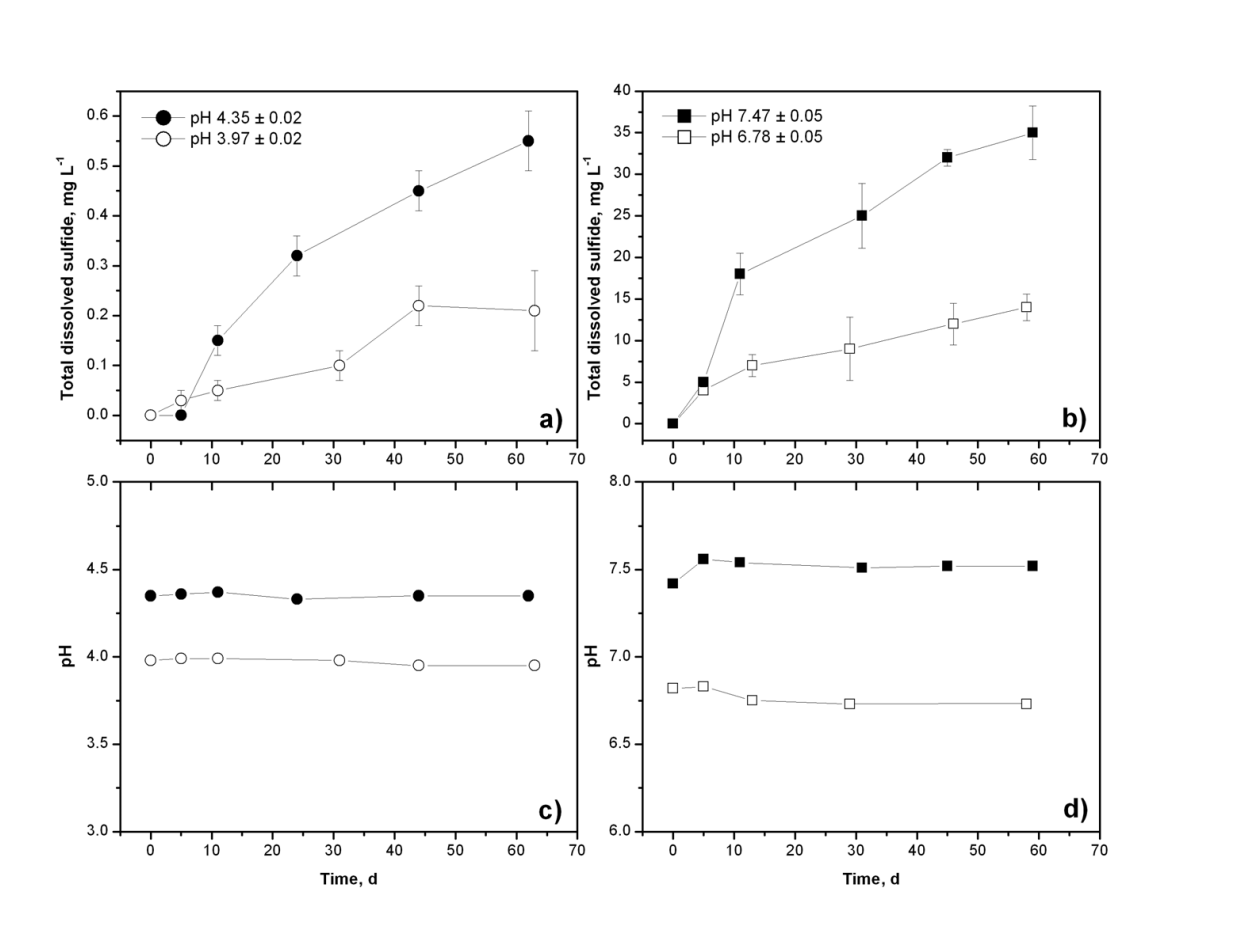
Results of microbial culturing experiments of Penn Mine sediments showing total dissolved sulfide concentrations and pH trends at low pH (near 4) and near-neutral pH.

Bacterial cultures were prepared in an anaerobic chamber using sediments collected from the Penn Mine workings. Sulfate reduction was visually evident after approximately 1 week based upon the appearance of black precipitates of iron sulfide. Low-pH and near-neutral pH cultures were next established by spiking the primary cultures into pH-adjusted growth media. Total dissolved sulfide and pH results of these experiments are shown in Figure 4. Sulfate reduction occurred at pH 4.0 to 4.35. Sulfide concentrations up to 0.48 mg L^-1 ^(15 μM) were observed after 60 days at pH 4.3. Higher sulfide concentrations were observed at higher pH (Figure 4b). Compared to results at pH 7, lower hydrogen sulfide concentrations at pH 4 could be related to increased toxicity of total dissolved sulfide to SRB. At pH below 7, the fully protonated form of hydrogen sulfide (H_2_SO) dominates, and this form is believed to be responsible for dissolved sulfide toxicity and inhibition of bacterial respiration [69]. Note that at pH 4, the sulfide concentration was insufficient to generate saturation with respect to FeS, at the ferrous iron concentration in the growth medium (~4 mg L^-1^) based on the observation that a black precipitate did not form.

**Figure 5:**
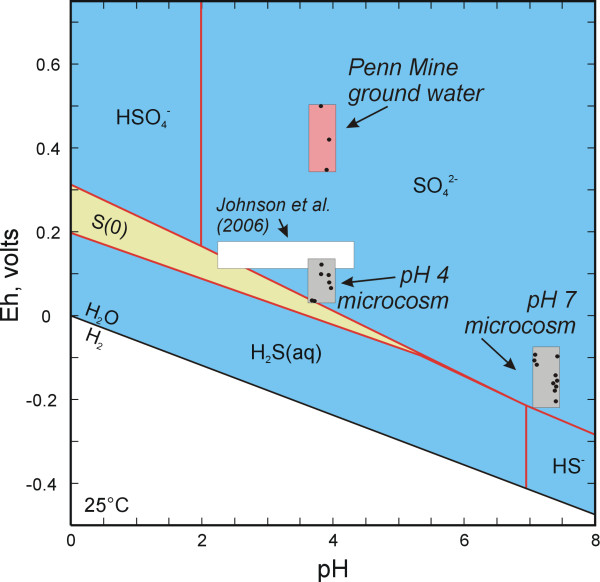
Eh-pH diagram (S-H_2_O system) for Penn Mine ground water and microcosm studies (Total S = 0.01 m).

At the conclusion of the experiments, the two low-pH and the two near-neutral-pH experiments were sampled and analyzed by DGGE. It is noteworthy that XRD analysis of the precipitate in the experiments revealed the presence of iron sulfide in the neutral-pH experiments and elemental sulfur in the low-pH experiments. The formation of iron sulfide in the near-neutral pH microcosms is not surprising; the development of black precipitates is often used as a visual indicator of sulfate reduction in bacterial cultures [70]. The formation of elemental sulfur in the low-pH microcosms suggests that oxidation of H_2_S(aq) occurred in the experiments. Inspection of an Eh-pH diagram for the S-H_2_O system indicates that the low-pH microcosms are in fact near the stability field for elemental sulfur (Figure 5). Therefore, at pH <5 there is a thermodynamic driving force for the precipitation of elemental sulfur from the microcosm solutions, i.e., the sulfur may not be a direct metabolic product. As a point of comparison, recent Eh-pH results from a low-pH sulfidogenic bioreactor study [14] are also shown on Figure 5. Note in all cases, the microcosm and bioreactor results fall in the aqueous sulfate field, but are close to the S(-II)-S(VI) boundary. Redox conditions in the Penn Mine ground water are more oxidizing compared to the microcosms (Figure 5); consequently, as previously noted it is possible that more reducing conditions were present in the mine workings below the sediment-water interface as compared to the overlying water column.

### DGGE of experimental cultures

**Figure 6:**
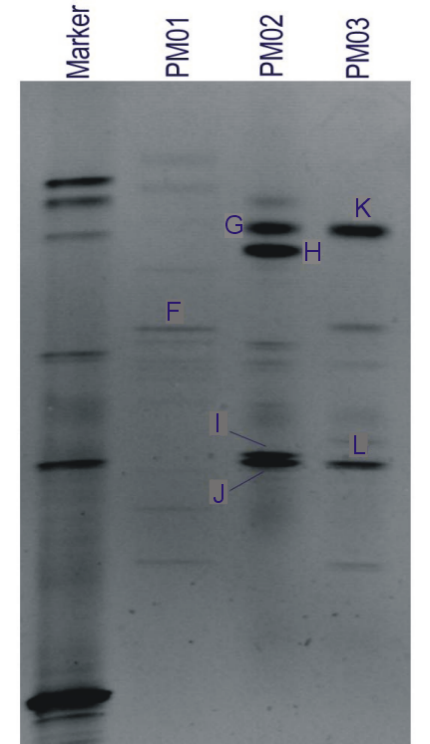
DGGE gel image of 16S rDNA fragments from microbial cultures derived from Penn Mine sediments with seven bands labeled (Table 4). PM01: water from culture experiments; PM02: low-pH cultures; PM03: near-neutral-pH cultures.

The DGGE profiles from the experimental cultures showed few prominent bands in each of the samples and few similarities between profiles (Figure 6). As expected from the observed trends in sulfide production shown in Figure 4, sulfur-reducing bacterial sequences were prominent in both samples. A sequence recovered from the water from experiment PM03 aligned with aerobic/facultatively aerobic bacterial sequences. The sequences recovered from the corresponding solid sample aligned with an aerobic bacterium, *Bacillus*. The results are summarized in Table 4 and discussed below.

**Table 4:** Sequence results from bands excised from DGGE of experimental cultures (Figure 6). Sequence identifications were performed using the BLASTN facility of the National Center for Biotechnology Information [47]. Similarity indices above 90% are considered excellent, 70–80% are good, and below 60% are considered to be unique sequences.

**Band**	**Closest Match**	**Similarity Index**	**Phylogemetoc Affiliation**
F	*Bacillus sp*. uncultured soil bacterium	100%	Bacillaceae
G, K	*Desulfosporosinus sp*. uncultured bacterium	92% 92%	Clostridia
H	*Desulfitobacterium sp*.	83%	Clostridia
I	uncultured bacterium	71%	Clostridia
J	uncultured bacterium	61%	Clostridia
L	*Burkholderia sp. Ralstonia sp*.	78%	Proteobacteria – Burkholderiaceae

Band F was very closely related to several sequences, all of which were classified in phylum Firmicutes and genus *Bacillus*, which have been recovered previously from samples from various studies (*e.g*. enzymatic oxidation of Mn(II), highly heat-resistant spores, and vanilla curing). Members of this genus are alkaliphilic in nature, and are generally aerobic, though some species are facultatively anaerobic and some species are known methylotrophs.

Bands G and K were closely related to published sequences classified in phylum Firmicutes, family Peptococcaceae, and genus *Desulfosporosinus*. The sequence most closely aligned with the Band G sequence had been recovered previously from a ZnS-producing biofilm dominated by sulfate-reducing bacteria in a subsurface acid mine drainage system [50], whereas the uncultured bacterium sequences were recovered from benzene-contaminated ground water [71]. Note that a bacterium closely related to *Desulfosporosinus *also was identified in the original Penn Mine sediment (Figure 3 and Table 3).

Band H also was closely affiliated with two sequences classified in phylum Firmicutes, family Peptococcaceae and genus *Desulfitobacterium*. The first sequence (accession number X95972) had been identified previously from a study looking at the influence of different electron donors and acceptors on dehalorespiration of perchloroethylene [68]. The second sequence (U40078) had been identified previously from a study of an anaerobic bacterium that reductively dechlorinates pentachlorophenol to 3-chlorophenol [68].

Band I was affiliated with a sequence recovered from the gut of a termite and classified as an uncultured bacterium in the phylum Firmicutes. There were slightly less similar matches to sequences classified in the Syntrophomonadaceae and Clostridiaceae families. Band J also was loosely associated with several sequences previously recovered from the gut of a termite and from oral cavities. Both sequences were classified in the phylum Firmicutes, Class Clostridia. Band L was aligned with two sequences, one of which had been identified previously from a study looking at the production of the antifungal compound phenylacetic acid by an antagonistic bacterium [72]. The recovered sequence was classified in the genus *Burkholderia*.

### Implications for natural attenuation

Metal sulfide precipitation as a remediation strategy has practical limits in moderately acidic environments (e.g., pH 3 to 5) because the solubility of most metal sulfides (but not arsenic) increases with decreasing pH. Consequently, with progressively lower pH values, higher dissolved sulfide concentrations would be needed in order to achieve remedial goals for metals (e.g., MCLs, maximum contaminant levels) via metal sulfide precipitation. Experimental data in Figure 4 suggest that low-pH sulfidogenesis results in comparatively low dissolved sulfide concentrations, perhaps related to increased toxicity of dissolved sulfide to SRB. Alternatively, elevated concentrations of dissolved metals can inhibit the growth of SRB [73]. Thus, an increase in mineral solubility, sulfide toxicity, or metal toxicity at low pH may restrict H_2_S production and result in limited natural attenuation. Thermochemical modeling was carried out to examine the solubility trends of zinc, cadmium, and copper at various pH conditions and total dissolved sulfide concentrations shown in Figure 4 (GEOCHEMIST'S WORKBENCH modeling package). At pH 4 and ΣH_2_S = 0.22 mg L^-1 ^(10^-5.2 ^molal), concentrations of Zn^2+^, Cd^2+^, and Cu^2+ ^at equilibrium with their respective freshly precipitated mineral sulfides were approximately 4 μg L^-1^, 0.01 μg L^-1^, and <0.01 ng L^-1^, respectively. Note that these concentrations are about 7 orders of magnitude higher than concentrations predicted at near-neutral pH and at the higher total dissolved sulfide concentrations (~10^-3.42 ^molal) shown in Figure 4b. Thermochemical model predictions, therefore, suggest that metal sulfide precipitation is a viable remediation strategy at moderately acidic conditions such as those encountered in the Penn Mine workings.

**Figure 7:**
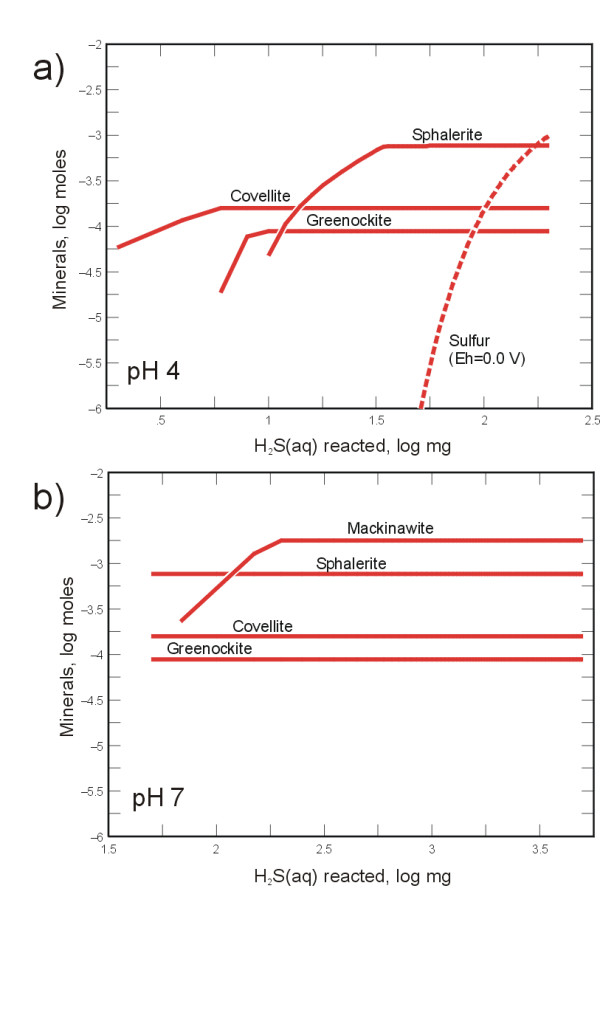
Reaction path modeling results showing trends in mineral precipitation as H_2_S(aq) is added to solutions containing Cd, Cu, and Zn. a) At pH 4, covellite (CuS), greenockite (CdS), and sphalerite (ZnS) precipitate, but mackinawite (FeS) remains undersaturated. b) At pH 7, all metal sulfides precipitate. The model was also run with the Eh fixed at 0.0 mV. At this condition elemental sulfur (dashed line) precipitation is favored at pH 4 following metal sulfide precipitation. Initial metals concentrations were Fe (150 mg L^-1^), Zn (100 mg L^-1^), Cd and Cu (15 mg L^-1^).

Reaction path modeling was carried out in order to examine these expected trends in more detail. The LLNL thermochemical database (thermo.com.V8.R6+.dat) was modified to include solubility data for mackinawite (FeS) [74]) and greenockite (CdS) [75]. Penn Mine ground water containing Zn^2+^, Cd^2+^, Cu^2+^, and Fe^2+ ^was titrated with H_2_S(aq) at constant pH. At pH 4, model results predict the initial precipitation of CuS after addition of as little as 0.1 μg H_2_S(aq) per liter of solution (Figure 7a). With increasing H_2_S(aq), CdS precipitation is followed by ZnS precipitation (Figure 7a). Precipitation of an iron sulfide is not predicted at pH 4, but precipitation of pyrite, troilite, and pyrrhotite was suppressed following Labrenz et al. [50] because it is unlikely that the rate of formation of these phases can compete with the formation rate of mackinawite. However, at pH 7 mackinawite formation is predicted following precipitation of ZnS (Figure 7b). In model runs with Eh constrained (0.0 to 0.15 V), elemental sulfur precipitation is predicted at pH 4 (dashed line on Figure 7a) but not at pH 7. These model results further suggest that metal sulfide precipitation can occur at low pH and help explain the trace metal ratios and the observed formation of elemental sulfur and mackinawite in the microcosms at pH 4 and 7, respectively.

## Conclusion

The data discussed here show evidence that bacterial sulfate reduction, metal attenuation, and thus, natural acid mine remediation, has been occurring to some degree in the Penn Mine. Water chemistry reveals: (1) preferential scavenging by H_2_S of Cu and Cd relative to Zn; (2) stable isotope ratios of ^34^S/^32^S and ^18^O/^16^O in dissolved SO_4 _were 2–3 ‰ heavier in the mine water relative to surface waters; and (3) reduction/oxidation conditions and dissolved gas chemistry consistent with the conditions to support anaerobic microbial processes such as sulfate reduction. SEM analysis showed the presence of ZnS precipitates. PLFA analyses showed a high biomass level in the Penn Mine sediments and the presence of lipid biomarkers indicative of SRB and other sulfur-reducing bacteria. DGGE analysis of the mine sediment indicates that the dominant bacteria are a diverse community composed of iron- and sulfur-reducing bacteria. Finally, cultures of the sediment material from the mine (1) were capable of producing dissolved sulfide at both near-neutral pH and at pH values near 4.0; and (2) formed precipitates of either FeS or elemental sulfur. DGGE analysis of the experimental cultures likewise were consistent with the presence of sulfur-reducing bacteria at pH 4.

These data suggest that engineered or natural bioremediation processes could remove certain trace metals from moderately acidic mine waters (pH around 4) if the appropriate conditions exist, namely (1) anaerobic conditions, (2) sufficient organic carbon, and (3) the presence of acid-tolerant sulfate-reducing bacteria, either naturally occurring or introduced. The present study indicates that metal attenuation occurs naturally in the pH-4 mine environment as a result of bacterial sulfate reduction. Our results imply that metal attenuation in natural, moderately acidic systems may be enhanced by maximizing microbial respiration through addition of organic carbon substrates (e.g., ethanol, compost, or manure).

## Supplementary Material

Additional File 1 Quality assurance and quality control for water analyses; image collected using down-hole camera during sampling of the Penn mine workings. Click here for file

Additional File 2 Table S1. Water-quality data for well GS-18, Penn Mine, California, 2001–02; Table S2. Statistical data for replicate water-quality samples, well GS-18, Penn Mine, California, 2002; Table S3. Quality assurance data for standard reference water samples. Click here for file

## References

[B1] Singer PC, Stumm W (1970). Acidic mine drainage: the rate-determining step. Science.

[B2] Nordstrom DK, Southam G (1970). Geomicrobiology of sulfide mineral oxidation. Reviews in Mineralogy.

[B3] Nordstrom DK, Alpers CN, Plumlee GS, Logsdon MJ (1999). Geochemistry of acid mine waters. The Environmental Geochemistry of Mineral Deposits Part A Processes, Methods, and Health Issues.

[B4] Edwards KJ, Bond PL, Gihring TM, Banfield JF (2000). An archaeal iron-oxidizing extreme acidophile important in acid mine drainage. Science.

[B5] Gammons CG, Metesh JJ, Snyder DM (2006). A survey of the geochemistry of flooded mine shaft water in Butte, Montana. Mine Water and the Environment.

[B6] Tuttle LH, Dugan PR, Randles CI (1969). Microbial sulfate reduction and its potential utility as an acid mine water pollution abatement procedure. Applied Microbiology.

[B7] Johnson DB (2003). Chemical and microbiological characteristics of mineral spoils and drainage waters at abandoned coal and metal sites. Water Air and Soil Pollution.

[B8] Fishbain S, Dillon JG, Gough HL, Stahl DA (2003). Linkage of high rates of sulfate reduction in Yellowstone hot springs to unique sequence types in the dissimilatory sulfate respiration pathway. Applied and Environmental Microbiology.

[B9] Willow MA, Cohen RRH (2003). pH, dissolved oxygen, and adsorption effects on metal removal in anaerobic bioreactors. Journal of Environmental Quality.

[B10] Kolmert A, Johnson DB (2001). Remediation of acidic waste waters using immobilised, acidophilic sulfate-reducing bacteria. Journal of Chemical Technology and Biotechnology.

[B11] Tsukamoto TK, Killion HA, Miller GC (2004). Column experiments for microbiological treatment of acid mine drainage: low-temperature, low-pH and matrix investigations. Water Research.

[B12] Kimura S, Hallberg KB, Johnson DB (2006). Sulfidogenesis in low pH (3.8–4.2) media by a mixed population of acidophilic bacteria. Biodegradation.

[B13] Kusel K, Roth U, Trinkwalter T, Peiffer S (2001). Effect of pH on the anaerobic microbial cycling of sulfur in mining-impacted freshwater lake sediments. Environmental and Experimental Botany.

[B14] Johnson DB, Sen AM, Kimura S, Rowe OF, Hallberg KB (2006). Novel biosulfidogenic system for selective recovery of metals from acidic leach liquors and waste streams. Mineral Processing and Extractive Metallurgy.

[B15] Sierra-Alvarez R, Karri S, Freeman S, Field JA (2006). Biological treatment of heavy metals in acid mine drainage using sulfate reducing bioreactors. Water Sci Technol.

[B16] Wendt-Potthoff K, Frommichen R, Herzsprung P, Koschorreck M (2002). Microbial Fe(III) reduction in acidic mining lake sediments after addition of an organic substrate and lime. Water Air and Soil Pollution Focus.

[B17] Koschorreck M, Frommichen R, Herzsprung P, Tittel J, Wendt-Potthoff K (2002). Functions of straw for *in-situ *remediation of acidic mining lakes. Water Air and Soil Pollution Focus.

[B18] Meier J, Costa R, Smalla K, Boehrer B, Wendt-Potthoff K (2005). Temperature dependence of Fe(III) and sulfate reduction rates and its effect on growth and composition of bacterial enrichments from an acidic pit lake neutralization experiment. Geobiology.

[B19] Koschorreck M, Bozau E, Frömmichen R, Geller W, Herzsprung P, Wendt-Potthoff K (2007). Processes at the sediment water interface after addition of organic matter and lime to an Acid Mine Pit Lake mesocosm. Environ Sci Technol.

[B20] Koschorreck M, Wendt-Potthoff K, Geller W (2003). Microbial sulfate reduction at low pH in sediments of an acidic lake in Argentina. Environmental Science and Technology.

[B21] Sen AM (2001). Ph D thesis.

[B22] Praharaj T, Fortin D (2004). Indicators of microbial sulfate reduction in acidic sulfide-rich mine tailings. Geomicrobiology Journal.

[B23] Kusel K (2003). Microbial cycling of iron and sulfur in acidic coal mining lake sediments. Water Air and Soil Pollution.

[B24] Roesler A, Gammons CH, Druschel GK, Oduro H, Poulson SR (2007). Geochemistry of flooded underground mine workings influenced by bacterial sulfate reduction. Aquatic Geochemistry.

[B25] Clark WB, Lydon PA (1962). Mines and Mineral Resources of Calveras County, California. California Division of Mines and Geology, County Report 2.

[B26] Peterson JA Geologic map of the Penn Mine, Calveras County, California. US Geological Survey Miscellaneous Field Studies Map MF-1797.

[B27] Bambic DG, Alpers CN, Green PG, Fanelli E, Silk WK (2006). Seasonal and spatial patterns of metals at a restored copper mine site: I. Stream copper and zinc. Environmental Pollution.

[B28] Hamlin SN, Alpers CN (1996). Hydrogeology and Geochemistry of Acid Mine Drainage in Ground Water in the Vicinity of Penn Mine and Camanche Reservoir, Calaveras County, California: Second-Year Summary, 1992–93. US Geological Survey Water-Resources Investigations Report 96–4257.

[B29] Alpers CN, Hamlin SN, Hunerlach MP (1999). Hydrogeology and Geochemistry of Acid Mine Drainage in Ground Water in the Vicinity of Penn Mine and Camanche Reservoir, California: Summary Report, 1993–95. US Geological Survey Water-Resources Investigations Report 96–4287.

[B30] Silk WK, Bambic DG, Green PG, O'Dell RE (2006). Seasonal and spatial patterns of metals at a restored copper mine site: II. Copper in riparian soils and *Bromus carinatus *shoots. Environmental Pollution.

[B31] Seal RR, Alpers CN, Rye RO, Alpers CN, Jambor JL, Nordstrom DK (2000). Stable isotope systematics of sulfate minerals. Sulfate Minerals: Crystallography, Geochemistry, and Environmental Significance Reviews in Mineralogy and Geochemistry.

[B32] Wilkin RT, Bischoff KJ (2006). Coulometric determination of total sulfur and reduced inorganic sulfur fractions in environmental samples. Talanta.

[B33] Huffman EWD (1977). Performance of a new automatic carbon dioxide coulometer. Microchemical Journal.

[B34] Brinton TL, Antweiler RC, Taylor HE (1995). Method for the determination of dissolved chloride, nitrate, and sulfate in natural water using ion chromatography. US Geological Survey Open File Report 95-426A.

[B35] McCleskey RB, Nordstrom DK, Ball JW (2003). Metal interferences and their removal prior to the determination of As(T) and As(III) in acid mine waters by hydride generation atomic absorption spectrometry. US Geological Survey Water-Resources Investigations Report 03–4117.

[B36] Stookey LL (1970). FerroZine – a new spectrophotometric reagent for iron. Analytical Chemistry.

[B37] McCleskey RB, Nordstrom DK, Naus CA (2004). Questa baseline and pre-mining water-quality investigation. 16. Quality assurance and quality control for water analyses. US Geological Survey Open File Report 2004-1341.

[B38] Epstein S, Mayeda T (1953). Variation of ^18^O content of waters from natural sources. Geochimica et Cosmochimica Acta.

[B39] Arak H, Brand WA (1995). A fully automated water/gas phase equilibration system for H and O isotope analysis. Application News, Finnigan MAT-252.

[B40] Fry BN, Brand W, Mersch FJ, Tholke K, Garritt R (1992). Automated-analysis system for coupled delta-C-13 and delta-N-15 measurements. Analytical Chemistry.

[B41] Kester CL, Rye RO, Johnson CA, Schwartz CC, Holmes CW (2001). Online sulfur isotope analysis of organic matter by direct combustion: preliminary results and potential applications. Isotopes in Environmental Health Studies.

[B42] Evans WC, White LD, Rapp JB (1988). Geochemistry of some gases in hydrothermal fluids from the southern Juan de Fuca Ridge. Journal of Geophysical Research.

[B43] Ringelberg DB, Townsend GT, DeWeerd KA, Suflita JM, White DC (1994). Detection of the anaerobic dechlorinating microorganism *Desulfomonile tiedjei *in environmental matrices by its signature lipopolysaccharide branch-long-chain hydroxy fatty acids. FEMS Microbiology Ecology.

[B44] Muyzer G, de Waal EC, Uitterlinden AG (1993). Profiling of complex microbial populations by denaturing gradient gel electrophoresis analysis of polymerase chain reaction-amplified genes coding for 16S rRNA. Appl Environ Microbiol.

[B45] Stephen JR, Chang YJ, Gan YD, Peacock A, Pfiffner S, Barcelona M, White DC, Macnaughton SJ (1999). Microbial characterization of a JP-4 fuel-contaminated site using a combined lipid biomarker/polymerase chain reaction-denaturing gradient gel electrophoresis (PCR-DGGE) based approach. Environmental Microbiology.

[B46] Röling WFM, Van Breukelen BM, Braster M, Goeltom MT, Groen J, Van Verseveld HW (2000). Analysis of microbial communities in a landfill leachate polluted aquifer using a new method for anaerobic physiological profiling and 16S rDNA based fingerprinting. Microbial Ecology.

[B47] National Center for Biotechnology Information. http://www.ncbi.nlm.nih.gov.

[B48] Altschul SF, Gish W, Miller W, Meyers EW, Lipman DJ (1990). Basic local alignment search tool. Journal of Molecular Biology.

[B49] Cline JD (1969). Spectrophotometric determination of hydrogen sulfide in natural waters. Limnology and Oceanography.

[B50] Labrenz M, Druschel GK, Thomsen-Ebert T, Gilbert B, Welch SA, Kemner KM, Logan GA, Summons RE, De Stasio G, Bond PS Lai B, Kelly SD, Banfield JF (2000). Formation of sphalerite (ZnS) deposits in natural biofilms of sulfate-reducing bacteria. Science.

[B51] Labrenz M, Banfield JF (2004). Sulfate-reducing bacteria-dominated biofilms that precipitate ZnS in a subsurface circumneutral-pH mine drainage system. Microbial Ecology.

[B52] Moreau JW, Webb RI, Banfield JF (2004). Ultrastructure, aggregation state, and crystal growth of biogenic nanocrystalline sphalerite and wurtzite. American Mineralogist.

[B53] Druschel GK, Labrenz M, Thomsen-Ebert T, Fowle DA, Banfield JF (2004). Geochemical modeling of ZnS in biofilms: An example of ore depositional processes. Economic Geology.

[B54] DiToro DM, Mahony JD, Hansen DJ, Scott JJ, Hicks MB, Mayr SM, Redmond MS (1990). Toxicity of cadmium in sediments: The role of acid volatile sulfide. Environmental Toxicology and Chemistry.

[B55] Lovley DR, Goodwin S (1988). Hydrogen concentrations as an indicator of the predominant terminal electron-accepting reactions in aquatic sediments. Geochimica et Cosmochimica Acta.

[B56] Hoefs J (1980). Stable Isotope Geochemistry.

[B57] Valley JW (1986). Stable isotope geochemistry of metamorphic rocks. Reviews in Mineralogy.

[B58] Shikazono NM, Hoshino M, Utada M, Nakata A, Ueda A (1998). Hydrothermal carbonates in altered wall rocks, at the Uwamuki Kuroko deposits, Japan. Mineralium Deposita.

[B59] Ohmoto H, Goldhaber M, Barnes HL (1997). Sulfur and carbon isotopes. Geochemistry of Hydrothermal Ore Deposits.

[B60] Pellicori DA, Gammons CH, Poulson SR (2005). Geochemistry and stable isotope composition of the Berkeley pit lake and surrounding mine waters, Butte, Montana. Applied Geochemistry.

[B61] Gammons CH, Poulson SR, Metesh JJ, Duaime TE, Henne AR (2003). Geochemistry and isotopic composition of H_2_S-rich flooded mine waters, Butte, Montana. Mine Water and the Environment.

[B62] White DC, Davis WM, Nickels JS, King JD, Bobbie RJ (1979). Determination of the sedimentary microbial biomass by extractable lipid phosphate. Oecologia.

[B63] Montero-Sanchez IC, Brimhall GH, Alpers CN, Swayze GA (2005). Characterization of waste rock associated with acid drainage at the Penn Mine, California by ground-based visible to short-wave infrared reflectance spectroscopy assisted by digital mapping. Chemical Geology.

[B64] Stackebrandt E, Sproer C, Rainey FA, Burghardt J, Päuker O, Hippe H (1997). Phylogenetic analysis of the genus Desulfotomaculum: evidence for the misclassification of Desulfotomaculum guttoideum and description of Desulfotomaculum orientis as Desulfosporosinus orientis gen. nov., comb. nov. Int J Syst Bacteriol.

[B65] Utkin I, Woese C, Wiegel J (1994). Isolation and characterization of Desulfitobacterium dehalogenans gen. nov., sp. nov., an anaerobic bacterium which reductively dechlorinates chlorophenolic compounds. Int J Syst Bacteriol.

[B66] Hengstmann U, Chin KJ, Janssen PH, Liesack W (1999). Comparative phylogenetic assignment of environmental sequences of genes encoding 16S rRNA and numerically abundant culturable bacteria from an anoxic rice paddy soil. Appl Environ Microbiol.

[B67] Röling WF, Van Breukelen BM, Braster M, Lin B, Van Verseveld HW (2001). Relationships between microbial community structure and hydrochemistry in a landfill leachate-polluted aquifer. Applied and Environmental Microbiology.

[B68] Nevin KP, Lovely DR (2002). Mechanisms for accessing insoluble Fe(III) oxide during dissimilatory Fe(III) reduction by Geothrix fermentans. Applied and Environmental Microbiology.

[B69] Moosa S, Harrison STL (2006). Product inhibition by sulphide species on biological sulphate reduction for the treatment of acid mine drainage. Hydrometallurgy.

[B70] Postgate JR (1984). The Sulphate-Reducing Bacteria.

[B71] Maymo-Gatell X, Chien Y, Gossett JM, Zinder SH (1997). Isolation of a bacterium that reductively dechlorinates tetrachloroethene to ethene. Science.

[B72] Gillis M, Tranvan V, Bardin R, Goor P, Hebbar A, Willems A, Segars P, Kerstens K, Heulin T, Fernadez M (1995). Polyphasic taxonomy in the genus *Burkholderia *leading to an amended description of the genus. International Journal of Systemic Bacteriology.

[B73] Poulson SR, Colberg PJS, Drever JI (1997). Toxicity of heavy metals (Ni, Zn) to *Desulfovibrio desulfuricans*. Geomicrobiology Journal.

[B74] Benning LG, Wilkin RT, Barnes HL (2000). Reaction pathways in the Fe-S system below 100°C. Chemical Geology.

[B75] Daskalakis KD, Helz GR (1992). Solubility of CdS (greenockite) in sulfidic waters at 25°C. Environmental Science and Technology.

