# Supplementary material for: Microbial sulfate reduction and metal attenuation in pH 4 acid mine water
Source: Geochem Trans. 2007 Oct 23;8:10. doi: 10.1186/1467-4866-8-10 (PMC2211471; doi:10.1186/1467-4866-8-10)
Supplement: Additional File 1 — Quality assurance and quality control for water analyses; image collected using down-hole camera during sampling of the Penn mine workings. [file 1467-4866-8-10-S1.doc]

**Supplemental Text for manuscript to be submitted to *Geochemical Transactions***

# *Church, C.D., et al., “*Microbial sulfate reduction and metal attenuation in pH 4 acid mine water*”*

**QUALITY ASSURANCE AND QUALITY CONTROL FOR WATER ANALYSES**

Several techniques were used to assure the quality of the analytical data for water samples. These techniques included calculation of speciated charge imbalance, analysis of a field blank, replicate analyses of environmental samples, and analysis of standard reference water samples (SRWS) prepared by the U.S. Geological Survey (USGS).

The speciated charge imbalance (C.I.) was calculated using the geochemical code WATEQ4F [1]. The C.I. was calculated as follows:

$C.I.(percent)=\frac{(sum cations-sum anions)}{(sum cations+sum anions)/2}\times100$ (1)

where “sum cations” and “sum anions” are in units of milliequivalents per liter. The charge imbalance calculated by WATEQ4F is twice the value that is typically reported for cation-anion balance because the term in the denominator is the average milliequivalents, rather than the sum of the milliequivalents. The C.I. is calculated on the speciated results rather than the raw analytical data because C.I. is dependent on speciation, especially for acidic solutions. The C.I. for the eight Penn Mine samples ranged from -12% to 11%, and the mean C.I. was -3%. The charge imbalance is primarily affected by the major ion chemistry and the errors are likely the result of analytics or unanalyzed constituents. Nordstrom et al. [2] considered samples analyses with charge imbalance of ±11 to be of sufficiently high quality for speciation calculations.

A field blank collected in Nov. 2001 was analyzed with the environmental samples to detect potential field or laboratory contamination. The field blank data are reported in table S1. With the exception of Fe, Cl, and SO_4_, all constituents in the field blank were below their method detection limits (table S1). Concentrations of Fe and SO_4_ were less than 0.5% of the lowest measured sample concentration, therefore contamination of these constituents is considered. The concentration of 1.0 mg/L of Cl in the field blank was only about twice the detection limit; however it is possible that concentrations of Cl in the environmental samples have a slight positive bias.

For each of the four environmental water samples collected during 2002, a replicate sample was collected for analysis of major cations and trace metals. Results of the replicate analyses are given in Table S2. Values of relative percent difference for environmental samples (RPD_E_) were computed using equation 2, where R1 and R2 represent the concentrations in the two replicate analyses.

(2)

For analytes below the method detection limits, RPD_E_ were not calculated.

RPD_E_ values (Table S2) were less than 15 % for all constituents in all four replicate samples. The mean of the absolute RPD_E_ values for each constituent was less than 10% for all constituents except Cr (11%). For stable isotopes, replicate results (Table S1) were interpreted in terms of absolute difference (AD, Table S2). Values of AD among replicates were within 0.1 per mill for all O and S isotope measurements and within 1 per mill for H isotopes (Table S2).

Several SRWS prepared by the USGS Water Resources Discipline Branch of Quality Services were analyzed as “unknowns” along with the environmental samples to check for accuracy. Standard reference water samples M6, AMW4, T159, and T163 were used to check the analytical methods for major cations and trace metals [3,4]. The compiled results for the SRWS include the number of analyses (n), concentrations, standard deviation or range of analyses, reported most probable value (MPV), F-pseudosigma (deviation), and the relative percent difference (Table S3). The relative percent difference between mean measured concentration and MPV for the SRWS (RPD_S_) was calculated using equation 3:

$\text{RPD}_{\text{S}}\text{ }=\frac{\text{(measured mean concentration}-\text{MPV)}}{\text{MPV}}\times100$ (3)

Analytical accuracy improves with increasing concentration; reduced accuracy near the method detection limit is the result of decreasing signal to noise ratio. Manganese (Mn) and boron (B) had high RPD_S_ for one or more SRWS. However, the environmental samples for this study had Mn concentrations ranging from 2.4 to 8.2 mg/L, more than 100 times higher than either SRWS. Therefore, the relatively high RPD_S_ for Mn on one SRWS is not a large concern in terms of study results. With regard to B, the RPD_S_ ranged from -12.9 to -34.4 for three SRWSs with MPVs from 0.011 to 0.032 mg/L. Concentrations of B in environmental samples from Penn Mine ranged from 0.11 to 2.8 mg/L, approximately 10 to 100 times greater than the range of B in the SRWSs. As with Mn, the results for B from the SRWSs are not a large concern with regard to the conclusions of this study. Additional information about the USGS SRWS program can be obtained at URL <http://bqs.usgs.gov/srs>.

**References Cited**

1. Ball, JW, Nordstrom, DK: **User's manual for WATEQ4F, with revised thermodynamic data base and test cases for calculating speciation of major, trace, and redox elements in natural waters.** U.S. Geological Survey Open-File Report 91-183, 1991, 189 p. <http://pubs.er.usgs.gov/usgspubs/ofr/ofr91183>.

2. Nordstrom, DK, McCleskey, RB, Hunt, AG, Naus, CA: **Questa baseline and pre-mining ground-water quality investigation. 14. Interpretation of ground-water geochemistry in catchments other than the Straight Creek Catchment, Red River Valley, Taos County, New Mexico, 2002-2003**. U.S. Geological Survey Scientific Investigations Report 2005-5050, 2005, 84 p.

3. Farrar, JW: **Results of the U.S. Geological Survey’s analytical evaluation program for standard reference samples distributed in October 1999.** U.S. Geological Survey Open-File Report 00-227, 2000, 143 p. <http://bqs.usgs.gov/srs/Report_Fall99.pdf>.

4. Connor, BF, Currier, JP, Woodworth, MT: **Results of the U.S. Geological Survey’s analytical evaluation program for standard reference samples distributed in October 2000.** U.S. Geological Survey Open-File Report 01-137, 2001, 116 p. <http://bqs.usgs.gov/srs/Report_Fall00.pdf>.

**Supplemental Figure**

Figure S1. Image collected using a down-hole camera during the sampling of sediments from the Penn Mine workings

**
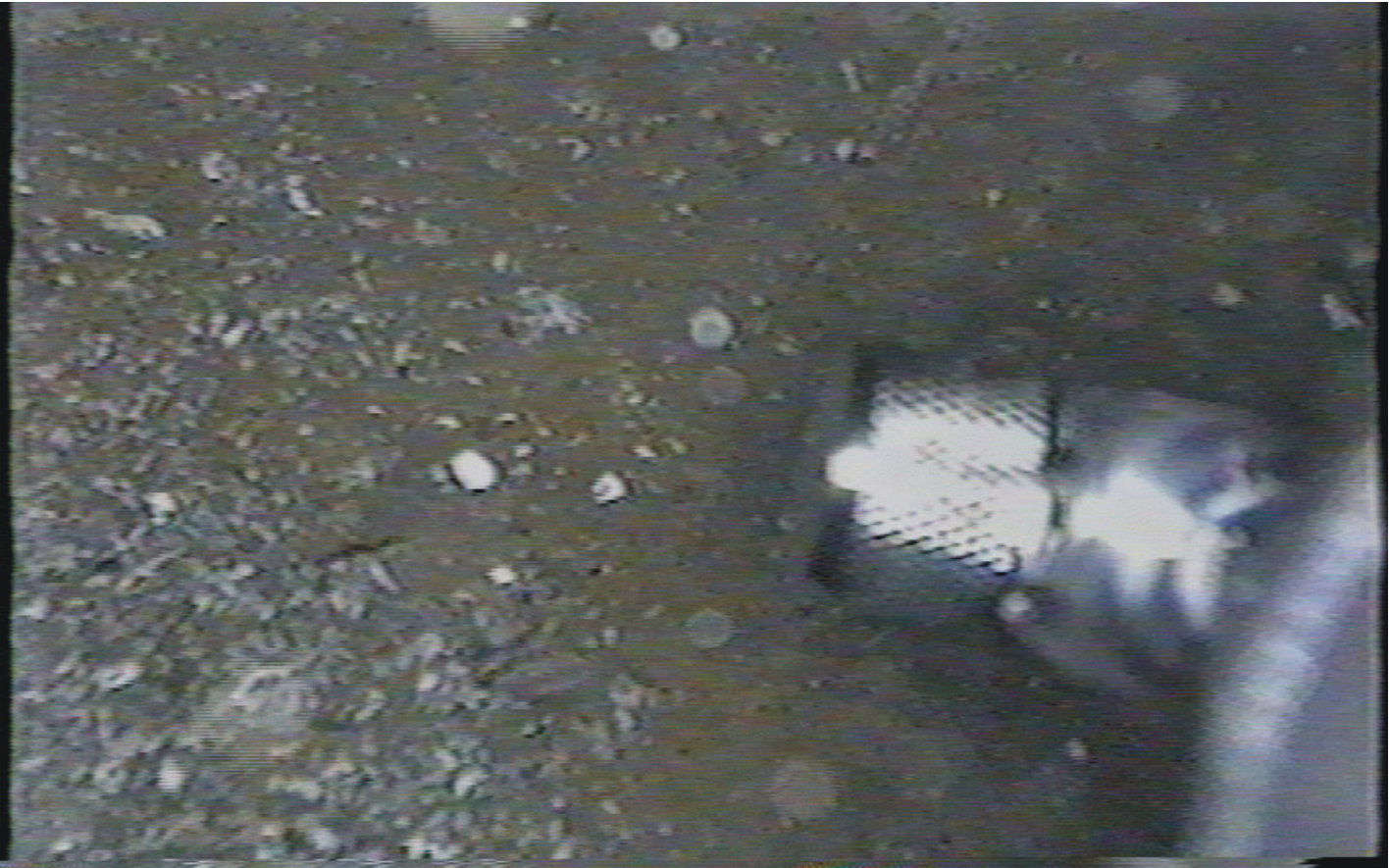
**

Sediments were recovered from flooded mine workings of the Penn Mine, a Cu-Zn mine abandoned since the early 1960s. Water chemistry, solid-phase characterization, and microbial characterization results all indicate conditions that support anaerobic processes such as sulfate reduction in the deep mine workings. At this site, sulfate-reducing bacteria play a role in attenuating metals at moderately low pH.
